# Narrow-diameter implants: Are they a predictable treatment option? A literature review

**DOI:** 10.4317/medoral.19306

**Published:** 2013-12-07

**Authors:** José L. Sierra-Sánchez, Amparo Martínez-González, Fernando García-Sala Bonmatí, José F. Mañes-Ferrer, Alejandro Brotons-Oliver

**Affiliations:** 1 Master in Advanced Oral Implantology Professor, Universidad Europea de Valencia; 2 Master in Advanced Oral Implantology Director, Universidad Europea de Valencia

## Abstract

Objective: To evaluate the predictability of narrow-diameter implants as a treatment option in routine clinical practice. A literature review was performed of studies reporting clinical results obtained with these implants. Survival rates, peri-implant bone loss and related complications were evaluated. The working hypothesis was that narrow-diameter implants offer clinical results similar to those obtained with implants of greater diameter. Material and Methods: A Medline-PubMed search covering the period between 2002 and 2012 was carried out. Studies published in English and with a follow-up period of at least 12 months were considered for inclusion. A manual search was also conducted in different journals with an important impact factor. Results: Twenty-one studies meeting the screening criteria were included in the literature review. A total of 2980 narrow-diameter implants placed in 1607 patients were analyzed. Conclusions: The results obtained from the literature indicate that narrow-diameter implants are a predictable treatment option, since they afford clinical results comparable to those obtained with implants of greater diameter.

** Key words:**Narrow implant, survival rate, peri-implant bone loss, related complications.

## Introduction

Treatment with dental implants offers a predictable solution for most situations seen in routine clinical practice. However, bone availability is often a limiting factor in planning our treatments.

A number of surgical techniques allow us to increase the available bone width, such as for example expansion with osteotomes (1), guided bone regeneration (2), autologous bone grafts (3), crestal expansion techniques (4), and osteogenic distraction (5,6).

Although these procedures offer good results in implantology, they are not without complications (7,8). The associated inconven-iences are increased morbidity, longer healing times, and infection secondary to wound dehiscence or membrane exposure (9,10). In patients with deficient crest width, the utilization of narrow-diameter implants therefore constitutes a technically more simple treatment alternative.

The definition of a narrow-diameter implant is subject to controversy. Although no universally accepted classification of implant diameters has been established to date, a narrow-diameter implant is generally taken to have a diameter of ? 3.0 mm and ? 3.5 mm. Some recent studies with narrow-diameter implants have reported implant success and survival rates similar to those obtained with greater diameter implants (11,12). However, few studies with prolonged periods of follow-up evaluating the predictability of these implants have been published.

The objective of the present literature review is to evaluate the predictability of narrow-diameter implants as an alternative to other technically more complex procedures, based on the survival rates, changes in peri-implant bone height and related complications.

## Material and Methods

- Search strategy

A Medline-PubMed search was conducted of studies published in English between January 2002 and June 2012 (both included), using the following MESH terms: “narrow implant”, “survival rate”, “peri-implant bone loss”, “related complications”. In order to minimize search bias, we also conducted a manual search of relevant articles published in four major implant journals with an important impact factor (International Journal of Oral & Maxillofacial Implants, Clinical Oral Implants Research, Journal of Periodontology, and Clinical Implant Dentistry and Related Research). The electronic and manual searches yielded fifty-one potentially relevant studies, based on the review of the corresponding abstracts. Following the full-text evaluation of these publications, only twenty-one studies were found to meet the inclusion criteria and were finally included in the review.

- Selection of studies and inclusion criteria

A single reviewer carried out the search. The variables of interest were implant survival, changes in peri-implant bone height, and related complications. Implant survival was defined as implant persistence in the mouth at the time of evaluation.

The studies included in the review were required to meet the following criteria:

o Full-text articles published in English in indexed journals between January 2002 and June 2012 (both included).

o Presentation of clinical results with implants of diameter ? 3.5 mm and ? of 3.0 mm.

o Systematic reviews, randomized clinical trials, and prospective or retrospective human cohort studies.

o A duration of follow-up of at least 12 months.

- Data extraction

All of the included studies were independently reviewed and analyzed. We collected data referred to the design of the studies (type of study, duration of follow-up, number of patients, number of implants, and type of edentulism) and the characteristics of treatment (type of implant, surgical technique employed, location of the implants, and type of prosthetic restoration). We also analyzed the variables reflecting the results of treatment (survival rate, peri-implant bone loss, associated complications [biological, prosthetic and aesthetic] and the results obtained with immediate loading).

## Results

- Variables associated to the study design ( Table 1)

**Table 1:**
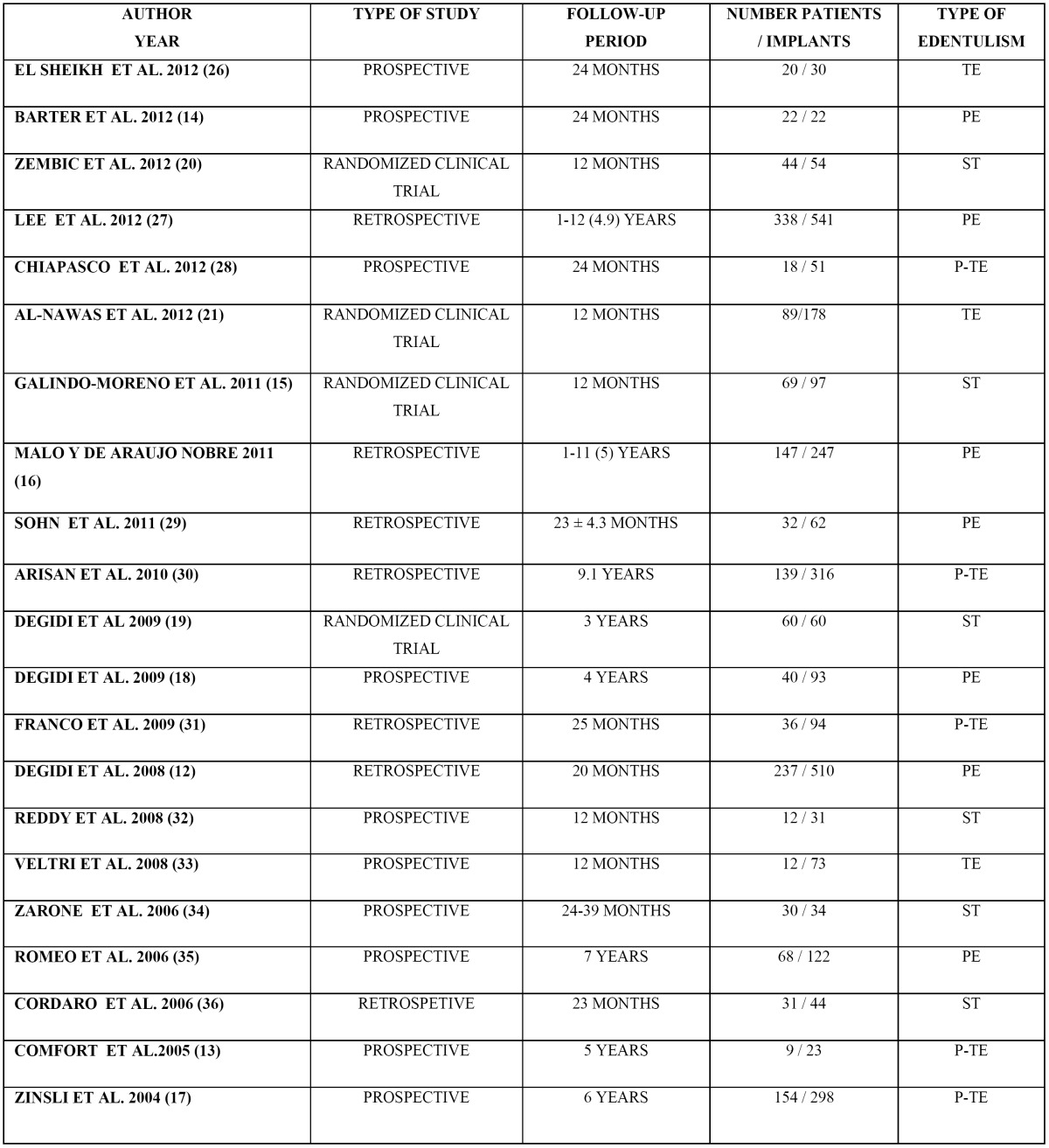
Variables associated to the study design ( ) Mean follow-up, TE: totally edentulous, PE: partially edentulous, P-TE: partially & totally edentuolous, ST: single-tooth.

The review included four randomized clinical trials (13-16), ten prospective studies (17-26) and seven retrospective studies (12,27-32). The follow-up periods ranged from 12 months to 12 years.

The twenty-one reviewed studies included a total of 1607 patients. Five studies involved over 100 patients (12,19,27,28,30). The patient age ranged from 13 to 87 years. Medically compromised subjects were excluded in all the studies. A total of 2980 implants were included in the review.

Regarding the type of edentulism, three studies included only totally edentulous individuals (16,21,24), five studies included both partially and totally edentulous patients (13,19,22,30,31), seven studies included partially edentulous cases (12,18,20,26-29), and six studies included only patients with a single missing tooth (13-15,23,25,32).

- Variables associated to the characteristics of treatment ( Table 2)

**Table 2:**
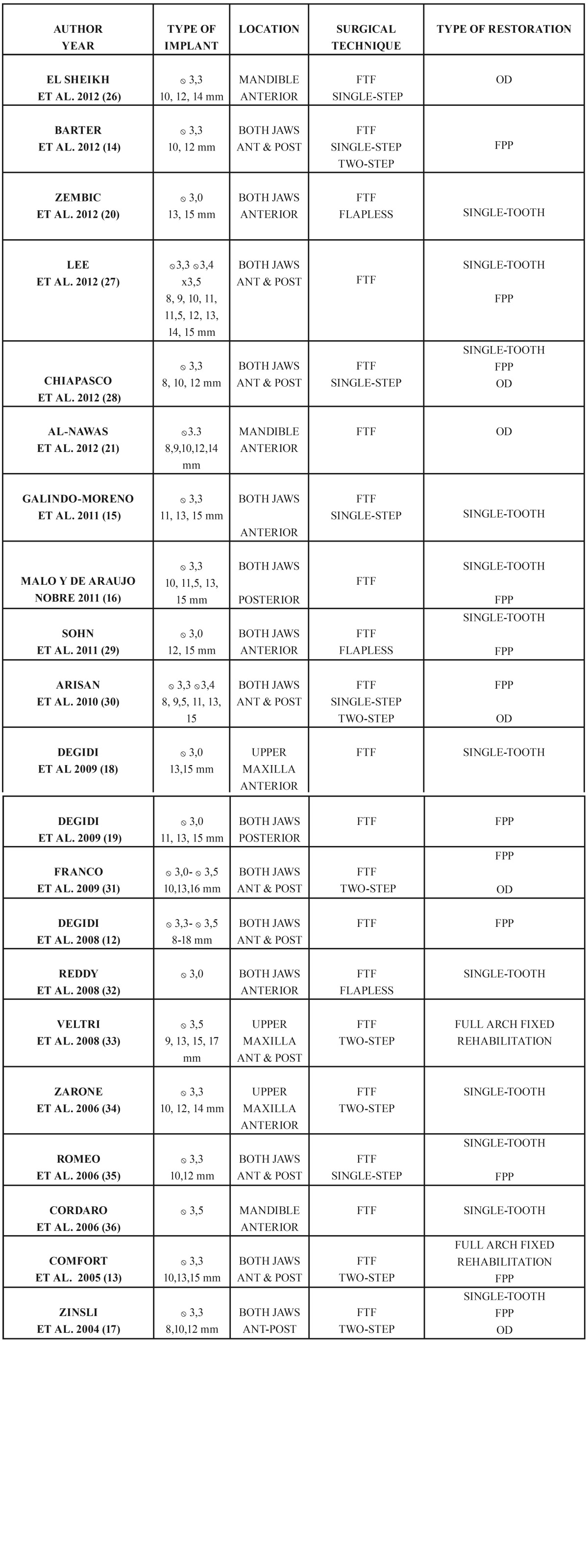
Variables associated to the characteristics of the treatment. OD: overdenture FPP: fixed partial prostheses ANT & POST: anterior & posterior FTF: full-thickness flap.

Use was made of implants with different designs and surface treatments. The diameter of the implants ranged between 3.0 and 3.5 mm, and the length between 8 and 18 mm. Apart from the study published by Comfort et al. (17), in which machined screw-shaped narrow implants (Bränemark System) were used, twenty studies described results corresponding to implants with different surface treatments (12-16,18-32). Two of these twenty studies used both machined implants and implants with surface treatments (27,28).

Regarding the location of the implants, nine studies presented results with implants placed only in the anterior zone (13-16,21,23,25,29,32), two studies reported results with implants positioned only in the posterior zone (20,27), and ten studies presented results corresponding to both the anterior and posterior zones (12,17-19,22,24,26,28,30,31).

Three studies presented results corresponding to implants positioned only in the mandible (16,21,32), another three studies included implants only in the upper maxilla (14,24,25), and fifteen studies included implants in both the upper maxilla and mandible (12-14,17-20,22,23,26-31).

Regarding the surgical technique employed, a full-thickness flap was raised for implant placement in all the studies (12-32). Nine studies used a single-step surgical protocol (12-15,20-22,26,27), five studies used a two-step protocol (17,19,24,25,31), two studies included both single and two-step surgical protocols (18,30), and three studies performed surgery involving the raising of a full-thickness flap without offering further details (16,28,29). Only three studies presented results comparing implants placed with a flapless technique versus the raising of a full-thickness flap (15,23,29).

Six of the reviewed studies included only single-tooth cases (13,14,15,23,25,26). Regarding the rest of the studies, three of them included only fixed partial prostheses (12,18,20), two studies presented only results corresponding to mandibular overdentures (16,21), and one study included only cases of full arch fixed rehabilitations (24). On the other hand, six studies presented results corresponding to both single-tooth cases and fixed partial prostheses (26,27,28,29-31), two studies presented results corresponding to single-tooth cases, fixed partial prostheses and overdentures (19,22), and one study presented results corresponding to both full arch fixed rehabilitations and fixed partial prostheses (17).

- Variables associated to the results of treatment ( Table 3)

**Table 3:**
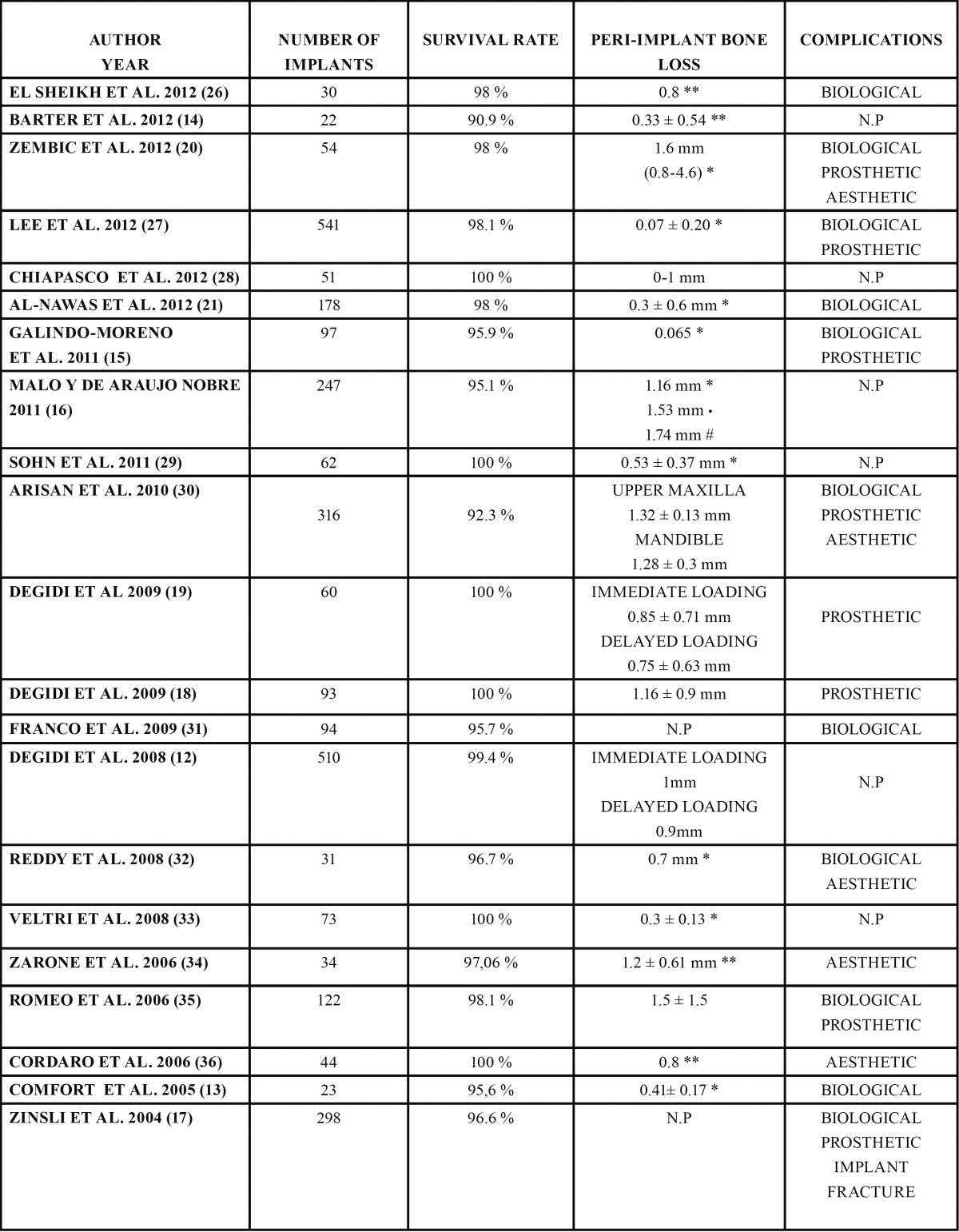
Variables associated to the results of the treatment * 1 year ** 2 years *** 3 years ? 5 years # 10 years N.P = NOT PUBLISHED.

The recorded implant survival rates were above 90% in all the studies. Six studies published a survival rate of 100% at the end of the follow-up period (14,20,22,24,29,32). The lowest survival rate (90.9%) corresponded to the study published by Barter et al. (18).

58 failures (implant loss) were recorded out of a total of 2980 implants. Only five studies (12,27,28,30,31) offered information on the length of the failed implants (43 implants). A larger number of failures were recorded with implants measuring ? 13 mm in length, compared with implants > 13 mm in length. The authors did not examine whether these data were statistically significant or not.

Nineteen studies measured changes in peri-implant bone height after implant loading (12-18,20-30,32). The values ranged be-tween 0.065 mm the first year according to Galindo-Moreno et al. (13) and 1.74 mm after a follow-up period of 10 years in the study published by Malo and de Araújo (27).

In relation to the recorded complications, eleven studies (13,15-17,19,21,23,26,28,30,31) registered biological complications. The latter were mainly related to a lack of implant osseointegration and infectious problems.

Prosthetic complications were registered in eight studies (13-15,19,20,26,28,30). Only the study published by Zinsli et al. (19) reported implant fractures. Specifically, the authors recorded two fractures after an observation period of less than two years in one case and more than six years in the other.

Other prosthetic complications of lesser importance associated to the use of narrow-diameter implants were also documented, such as screw loosening, prostheses decementation, screw fracture or prostheses fracture.

Five studies presented results referred to aesthetic complications (15,23,25,30,32). These problems mainly involved a poor aesthetic outcome of the definitive restoration or insufficient filling of the interproximal papilla.

Lastly, as regards of the results obtained with immediate loading, only five studies (12,14,15,20,27) offered information compar-ing an immediate loading protocol and a delayed loading protocol. In 2008, Malo and de Araujo (27) published a retrospective study on 3.3 mm implants placed in posterior areas and rehabilitated following an immediate loading protocol. The recorded implant survival rate was 95.5% after 9 years of follow-up. That same year, Degidi et al. (12) published a study comparing delayed and immediate loading in narrow-diameter implants placed in both anterior and posterior zones. The reported survival rate was 99.4% after a mean follow-up of 20 months. In 2009, the same authors (20) reported a 100% survival rate with 3.0 mm implants. In this study the authors treated patients with partially edentulous posterior areas rehabilitated with an immediate loading protocol followed-up on for 48 months. Likewise in 2009, Degidi et al. (14) published another study comparing peri-implant bone loss and probing depth between the two protocols. In this study, involving a follow-up period of 36 months, the differences were not statistically significant. In 2011, Zembic et al. (15) reported a survival rate of 98% for single 3.0 mm implants subjected to immediate loading and followed-up on for one year.

## Discussion

Some studies have found the survival of narrow-diameter implants (3.0 - 3.5 mm range) to be comparable to those obtained with standard-diameter implants (12,19). This review only included four randomized clinical trials supporting such performance (13-16).

Thirteen of the twenty-one studies included in the review presented a mean follow-up period of at least 24 months (14,17-22,25-28,30,31). The patient sample was quite large and included both totally and partially edentulous individuals. Due to the great variety of implants analyzed and the high survival rates recorded, it is difficult to establish a relationship between the characteristics of the different surfaces and implant survival. Regarding length, the failure rate tended to increase when using implants measuring ? 13 mm in length, compared with longer implants. The statistical significance of these results could not be analyzed.

In 2006, Cardaropoli et al. (33) reported a mean interproximal bone loss of 1.5 mm during the first year for standard-diameter implants. Other authors, based on finite elements analysis, found that an increase in implant diameter exerts a greater effect in terms of the reduction of stress transmission than an increase in implant length (34).

The possibility is therefore raised that smaller-diameter implants are associated to greater bone loss after functional loading. The results obtained in this review contradict this idea, since thirteen of the reviewed studies recorded a mean bone loss of ? 1 mm (12-14,16-18,21-24,28,29,32). The greatest peri-implant bone loss value was 1.74 mm, reported in a study published by Malo and de Araujo, with a follow-up period of 10 years (27).

Independently of the surgical technique used, good results were obtained with narrow-diameter implants placed in both the anterior and the posterior areas of both jaws.

Favorable results were also obtained with narrow-diameter implants supporting different types of prosthetic restorations, some of which implied increased biomechanical demands upon the implants.

Although some authors have related the use of narrow-diameter implants with an increased risk of implant fracture (35,36), no such association was observed in this review. Indeed, only two fractures were recorded out of a total of 2980 implants. In any case, these results are probably conditioned by the study designs involved.

Since the need for bone grafts or regeneration techniques was obviated, the incidence of biological complications was relatively low. The recorded prosthetic complications were generally few and easy to resolve. Very little information was obtained on the aesthetic complications associated to the use of narrow-diameter implants.

Only five of the twenty-one studies included in the review contributed information on the results obtained with narrow-diameter implants subjected to immediate loading (12,14,15,20,27). The follow-up periods in these studies were 12 months, 20 months (mean follow-up), 36 months, 48 months and 9 years. Good results (with implant survival rates of over 95%) were observed in all of them. However, since the publications were so few, further studies involving longer periods of follow-up are needed in order to confirm these results.

Different factors may have influenced the results obtained. Firstly, most of the studies excluded smokers, patients with bruxism and medically compromised subjects from the analysis of results. Secondly, bone quality - which conditions primary implant stability - and the experience of the clinician, may have exerted a decisive influence.

## Conclusion

Despite the limitations inherent to reviews of this kind, the results obtained appear to confirm the idea that treatment with narrow-diameter implants offers clinical results in terms of implant survival, peri-implant bone loss and associated complications similar to those of treatment with implants of greater diameter. Further studies are needed, with longer follow-up periods, in order to confirm these conclusions.
